# Investigating the aftermath of the Türkiye 2023 earthquake: exploring post-disaster uncertainty among Syrian migrants using social network analysis with public health approach

**DOI:** 10.3389/fpubh.2023.1204589

**Published:** 2023-07-27

**Authors:** Gamze Aktuna, Şevkat Bahar-Özvarış

**Affiliations:** ^1^Department of Public Health, Faculty of Medicine, Hacettepe University, Ankara, Türkiye; ^2^Department of Infectious Disease Epidemiology, Robert Koch Institute (RKI), Berlin, Germany; ^3^Institute of Public Health, Hacettepe University, Ankara, Türkiye

**Keywords:** uncertainty, migrants and refugees, earthquake, disaster, social network analysis, content analysis, Turkey, Syrian

## Abstract

**Objectives:**

On February 6th, 2023, a doublet earthquake struck Türkiye, impacting more than 15 million people including migrants, and resulting in over 50,000 deaths. The Syrian migrants experience multiple uncertainties in their daily lives which are further compounded by multifaceted challenges of the post-disaster environment. Social media was used intensively and with impunity in this environment and thereby provides a window into the explicit and implicit dynamics of daily life after a disaster. We aimed to explore how a post-disaster environment potentially generates new uncertainties or exacerbating pre-existing ones for migrants through social media analysis with an indirect perspective, in the context of 2023-Earthquake in Türkiye and Syrian migrants.

**Methods:**

Social network analysis was used to analyze Twitter-data with the hashtags ‘Syrian’ and ‘earthquake’ during a 10-day period beginning on March 22nd, 2023. We calculated network metrics, including degree-values and betweenness-centrality and clustered the network to understand groups. We analyzed a combination of 27 tweets with summative content analysis using a text analysis tool, to identify the most frequently used words. We identified the main points of each tweet and assessed these as possible contributors to post-disaster uncertainty among migrants by using inductive reasoning.

**Results:**

There were 1918 Twitter users, 274 tweets, 124 replies and 1726 mentions. Discussions about Syrian migrants and earthquakes were established across various groups (*n*_groups(edges > 15)_ = 16). Certain users had a greater influence on the overall network. The nine most frequently used words were included under uncertainty-related category (*n*_most_frequently_used_words_ = 20); ‘aid, vote, house, citizen, Afghan, illegal, children, border, and leave’. Nine main points were identified as possible post-disaster uncertainties among migrants.

**Conclusion:**

The post-disaster environment has the potential to exacerbate existing uncertainties, such as being an undocumented migrant, concerns about deportation and housing, being or having a child, inequality of rights between being a citizen and non-citizen, being in minority within minority, political climate of the host nation and access to education or to generate new ones such equitable distribution of aid, which can lead to poor health outcomes. Recognizing the possible post-disaster uncertainties among migrants and addressing probable underlying factors might help to build more resilient and healthy communities.

## Introduction

On February 6th, 2023, Türkiye’s eastern region was hit by a catastrophic doublet earthquake, which heavily affected more than ten cities with a total population of over 15 million, including Syrian migrants (1). The Minister of Interior Affairs of Türkiye stated on April 5th that over 50,000 people had lost their lives (2), and the President of the Disaster and Emergency Management Presidency of Türkiye declared on March 20th that over 100,000 people were injured (3). Most casualties were reported in the Turkish cities of Adıyaman, Gaziantep, Hatay, and Kahramanmaraş (4). At this juncture, it is crucial to be aware of the challenges associated with obtaining updated national statistics related to the earthquake. Numerous ministries and organizations, both national and international, have joined forces to respond to the devastating earthquakes in Türkiye. The Ministry of Justice, Ministry of Family and Social Policies, Ministry of Foreign Affairs, and Ministry of Health, among others, have coordinated efforts to provide aid to those affected. These include setting up coordination centers and field hospitals, offering psychosocial support, providing education and training for all age groups, establishing youth centers, constructing new dormitories for students, caring for livestock, offering debt restructuring, and credit to small businesses, investigating damaged buildings, relocating prisoners and executing several other post-disaster response efforts (5).

Approximately 1.7 million Syrian migrants live in these Eastern Türkiye provinces affected by the earthquake (6). On March 13th, the Minister of Interior Affairs disclosed that more than 6,000 foreign nationals, predominantly Syrians, had lost their lives (7). The latter fact was further corroborated by a distinct media outlet, which asserted that approximately 50 percent of the bodies of 6,000 decedents were repatriated to Syria (8, 9). Additionally, the Minister of National Defense announced that approximately 42,000 Syrians had returned to Syria following the earthquakes (10). Many governmental authorities, United Nations agencies, and nongovernmental organizations (NGO) working with Syrian migrants contributed to post-disaster response and recovery activities in an inclusive manner, conducting needs assessments and providing humanitarian relief items including tents, food, water, diapers, sanitary pads, wet wipes, and sleeping bags (11). Governmental bodies have continuously implemented interventions to minimize the effect of the earthquakes on Syrian migrants. An initial announcement was made permitting migrants registered in the affected provinces to relocate to another city without a travel permit for 90 days. However, the Directorate General of Migration Management later imposed limitations on eligible cities and duration (12). Notwithstanding their exposure to the same disaster as citizens, this vulnerable group, who lacks the right to unrestricted mobility, confronts a range of obstacles across the disaster’s response and recovery phases. As per a report by another non-governmental organization, Syrian migrants encountered multiple challenges subsequent to the earthquake, such as discriminatory language, insufficient availability of housing, and restricted access to aid. The report proposes various possible remedies, including taking a firm stance against defamatory and discriminatory news targeting migrants, and ensuring a robust dissemination of accurate information (13).

Migrants, who already struggle with numerous social determinants of health in normal times, face multidimensional challenges in times of disaster. Disasters often exacerbate existing inequalities and make it even more difficult for vulnerable groups to access healthcare and other essential services (14). For migrants, these challenges include language barriers, lack of access to information, limited social networks, substandard living and discrimination (15). In addition, disasters can result in new displacement, loss of livelihood, and other economic hardships that can further impact health outcomes (16). The intricate interplay of different factors highlights the need for tailored interventions and policies aimed at minimizing the effects of disasters, particularly in vulnerable groups, such as migrants (17).

Behind all these post-disaster environment challenges is another phenomenon that is difficult to quantify and characterize, namely uncertainty. Uncertainty can manifest in various facets of the pre-disaster period or emerge as a consequence of the disaster, subsequently exacerbating the situation. Uncertainty refers to a subjective state of cognitive and emotional unease characterized by a lack of predictability, clarity, or confidence about future events, outcomes, or information. It is a fundamental aspect of human cognition and decision-making, stemming from the inherent limitations of knowledge, information gaps, and complexity of the world. It involves a range of psychological and emotional experiences, including doubt, ambiguity, and insecurity (18–23). The immediate aftermath of a mass casualty event like disaster is marked by a period of heightened uncertainty (24). Disasters frequently affect people and communities profoundly and permanently, bringing uncertainty and interruptions to many aspects of daily life (25). Chaos and unpredictability are frequent features of post-disaster environment, that can increase stress and uncertainty in affected individuals (26). People may find it difficult to re-establish a sense of security and control in their lives following a disaster, which can cause a protracted period of uncertainty (27).

The experience of uncertainty has multifaceted impacts on human health and well-being, irrespective of the underlying condition, causing to feel anxious, helpless, and depressed (28, 29). During periods of uncertainty, the brain’s metabolic demands escalate, and if the state of uncertainty endures, it may lead to a cerebral energy crisis that can result in burdening the individual by ‘allostatic load’ contributing to systemic and brain malfunction (impaired memory, atherogenesis, diabetes and subsequent cardio- and cerebrovascular events) (30). Furthermore, apart from its impact on physical well-being, uncertainty is a significant determinant of poor mental health outcomes. Numerous studies have put forth the notion that the subjective experience of uncertainty has an adverse impact on the mental well-being of individuals across diverse environment (31–33).

Uncertainty becomes a complex interwoven structure influenced by numerous external elements when incorporated within the framework of migration and post-disaster environment. Current academic literature reveals a lack of publications adopting a comprehensive approach that brings together the topics of uncertainty, migration, and disasters. However, existing literature does provide insights into the uncertainties experienced by migrants. The causes for uncertainty range from access to education and healthcare, exposure to violence, and so on (34, 35). The emotional instability experienced by migrants is a consequence of the complex interplay of political and social factors linked to their displacement, resulting in a pervasive sense of uncertainty that significantly affects their overall well-being and mental health (36, 37). Disasters often have a profound impact on the population living in affected areas. These events can lead to significant physical and emotional trauma as well as economic and social disruption. In the aftermath of disasters, the resilience of affected populations can be severely tested and their ability to cope with stress and uncertainty can be compromised. This can lead to increased tensions and conflicts, particularly in situations where resources are scarce or competition for resources is high (38, 39). In regions with large refugee and migrant populations, the impact of disasters can be even more significant. This information together with the potential of disasters to contribute uncertainty highlight the complexities and challenges regarding uncertainties faced by migrants in post-disaster environment.

Examining uncertainty as a phenomenon, particularly in relation to the long-term effects of disasters on migrants, might help promote adequate public health interventions for migrants and highlight a number of background factors that may have subtle but significant dual impacts on health. However, the comprehension of intricate and multifaceted concepts as uncertainty, that cannot be directly quantified, necessitates methodologies that diverge significantly from the most commonly used public health research methodology. Fortunately, the field of public health is characterized by its dynamic nature, as it consistently integrates diverse disciplinary perspectives and employs a variety of methodological approaches (40). One such approach that is gaining prominence in today’s increasingly interconnected social world is social network analysis (41). As an open access and continuously updating source of data, social media analysis is proving to be a valuable tool in the field of public health (42, 43), particularly in crises situations with limited data collection opportunities (44). It also takes on being a useful tool as a transparent window into the explicit and implicit dynamics of daily life after a disaster, while been used intensively and with impunity. We presume this method enables an indirect perspective to access discussions in where possible post-disaster uncertainties among migrants are hidden. We aimed to explore how a post-disaster environment potentially generates new uncertainties or exacerbating pre-existing ones for migrants through social media analysis with an indirect perspective, in the context of 2023-Earthquake in Türkiye and Syrian migrants. We attempted to understand the research subject (possible post-disaster uncertainties among migrants) through analyzing social media content limited by hashtags and generated in the host country’s language and interpretation of these media content regarding the recent literature and current sociopolitical atmosphere in the context of 2023-Earthquake in Türkiye and Syrian migrants.

## Methods

### Social network and summative content analysis

We employed a social network analysis (SNA) methodology using Twitter data in this research to serve as a transparent window into the explicit and implicit dynamics of daily life after a disaster and consolidated with content analysis. Our methodological steps were to _(1)_construct a network graph based on user interactions to understand the social network dynamics in post-disaster environment in order to serve as a baseline for content analysis, and _(2)_conduct summative content analysis, wherein the analysis commenced with a word count (45) and later the main point of tweets were identified with using an inductive reasoning in order to explore the research subject.

### Data collection

To gather and focus the research related data, we searched for public tweets related to our research question containing the hashtags in Turkish ‘Syrian (*Suriyeli)’ and ‘earthquake (*deprem)’ during a 10-day period beginning on March 22nd, 2023. We defined our network in this research as a group of Twitter users who use these two hashtags and some of whom engage in some form of interaction with each other. In our network, there were 2,126 different type of Twitter actions including tweets and any kind of interactions which refer to the dynamic and interactive engagement between users on the Twitter platform, involving various forms of communication, such as retweets, replies and mentions. We utilized Twitter’s application programming interface (API) to collect network data, which provided us information including the tweet’s author, content (tweet), time, and any mentions or replies. The inclusion criteria for tweets were having both hashtags and there were no exclusion criteria for the social network analysis.

### Data analysis

We analyzed the social network graph utilizing NodeXL, a validated methodology used in previous research (46).

(1) We calculated several social network analysis metrics, such as in/out-degree and Eigen vector to understand the structural properties of the social network, including the flow of information, the importance of nodes, and their influence within the network.
(2) We identified influential users by using the betweenness centrality metric, designed to rank users according to their position in a network (47).
(3) The Clauset-Newman-Moore algorithm is employed for clustering the vertices of the social network to identify groups of closely connected nodes within the social network, allowing us to analyze interactions within these distinct subgroups.
(4) We used two stages to include tweets in content analysis.
(4a) The first stage was to directly include all tweets within the most interacted groups determined based on having a larger number of edges exceeding 15, with a second check by the researchers to ensure that there were no meaningless tweets, such as hashtags-only or nonsense wording. This stage ensured to capture tweets that are highly engaged with and have the potential to reflect important discussions or trends within the social network.
(4b) The second stage was identifying tweets within our dataset that could potentially provide valuable insights to address the research question, irrespective of their inclusion in the most interacted groups. To achieve this, the researchers removed all tweets posted by these most interacted groups from the entire tweet dataset, examined the remaining tweets, and selected those for inclusion in the summative content analysis by consensus. This second stage safeguarded not to exclude any content that may be valuable to the research question.
(5) We combined all tweets from most interacted groups(*n*_groups(edges > 15)_ = 16) and 15 tweets identified from smaller groups by the second-scanning of the entire dataset. This step compiled a comprehensive dataset of tweets that represent both highly engaging conversations and additional valuable perspectives.
(6) After removing duplicates, we analyzed a combined text of 27 tweets using Voyant Tools, an open-source web-based text analysis tool (48), to identify the most 20 frequently used words. By examining the frequency of words we targeted to grasp common themes, topics, or sentiments expressed in the tweets and gain a deeper understanding of the content being shared.
(7) The researchers assessed the place of most frequently used words in the combined text and aimed to find the tweets that including these most frequently used words to identify recurring patterns or key themes that emerge from the tweets, providing insights into the underlying common message or focus of the circulated conversations. In our research, each of 27 tweets included at least one of these most frequently used words.
(8) Tweets were read and re-read, and the main point of each tweet was identified and examined in the context of the research question based on the literature and qualifications of the researchers working in this field.
(9) Concurrently, the most frequently used words in these tweets were re-evaluated and it was consented that some words did not have a direct link to the research question and given that the frequent use of these words would not lead to any induction.
(10) The top 20 most frequently used words were then sorted into two categories to provide a structured framework for further analysis and interpretation.
(10a) Material-related category, in which most frequently used words have no connotation or literal meaning associated with main points of tweets (*n*_material-related_words_ = 11) and
(10b) Meaning-related category in which each of these most frequently used words plays a representing keyword role of the main point of tweet.
(11) At that point, the researchers decided to proceed with the inductive reasoning process by utilizing tweets including words belonging to the meaning-related category. The decision to analyze tweets included in this category was driven by the goal of capturing dominant meaningful themes and discussions related to the research subject, ensuring a comprehensive and utmost representative analysis of the prevailing sentiments and key topics within the available data.
(12) In line with recent literature, current events, and relevant sociopolitical issues we performed inductive reasoning, to assess the main point of each tweet as one potential way how post-disaster environment might generate new uncertainties or exacerbate pre-existing ones for migrants. Through the process of inductive reasoning, we analyzed the available scientific knowledge to draw logical conclusions and uncover probable insights regarding potential ways.
(13) And we designated these potential ways as the research subject, entitled ‘possible post-disaster uncertainties among migrants’.
(14) Meaning-related category was renamed to uncertainty-related category because every tweet that contained the words in this category was agreed to be linked to the research subject (*n*_uncertainty-related_words_ = 9). Some main points of tweets were identified as having almost identical phrasing to the most frequently used uncertainty-related words. Diverse phrases of main points have also been identified owing to the varying connotations of uncertainty-related words and their neutral semantic structure. Each of these main points of tweets represents possible post-disaster uncertainty among migrants (*n*_main_points_ = 9).

### Quality assurance

Quality assurance was performed in reporting this research based on the Social Networks in Health Research (SoNHR) guideline, which was published as a preprint on April 25th, 2023 (49).

### Linkage with Ph.D. dissertation

This research serves as a supplementary study to a public health dissertation, wherein an uncertainty scale is developed for migrants, and the relationship between uncertainty and the health status of adult Syrian migrants is analyzed, in order to enhance the comprehension of phenomenon in different settings. The theoretical framework of this dissertation research series is rooted in the notion that uncertainty may function as a social determinant of migrants’ health and understanding this phenomenon could help tailor public health interventions to the specific needs of migrants, focusing on the increasing momentum of uncertainty in migration and its evidence-based impact on poor health outcomes.

## Results

**Figure 1:**
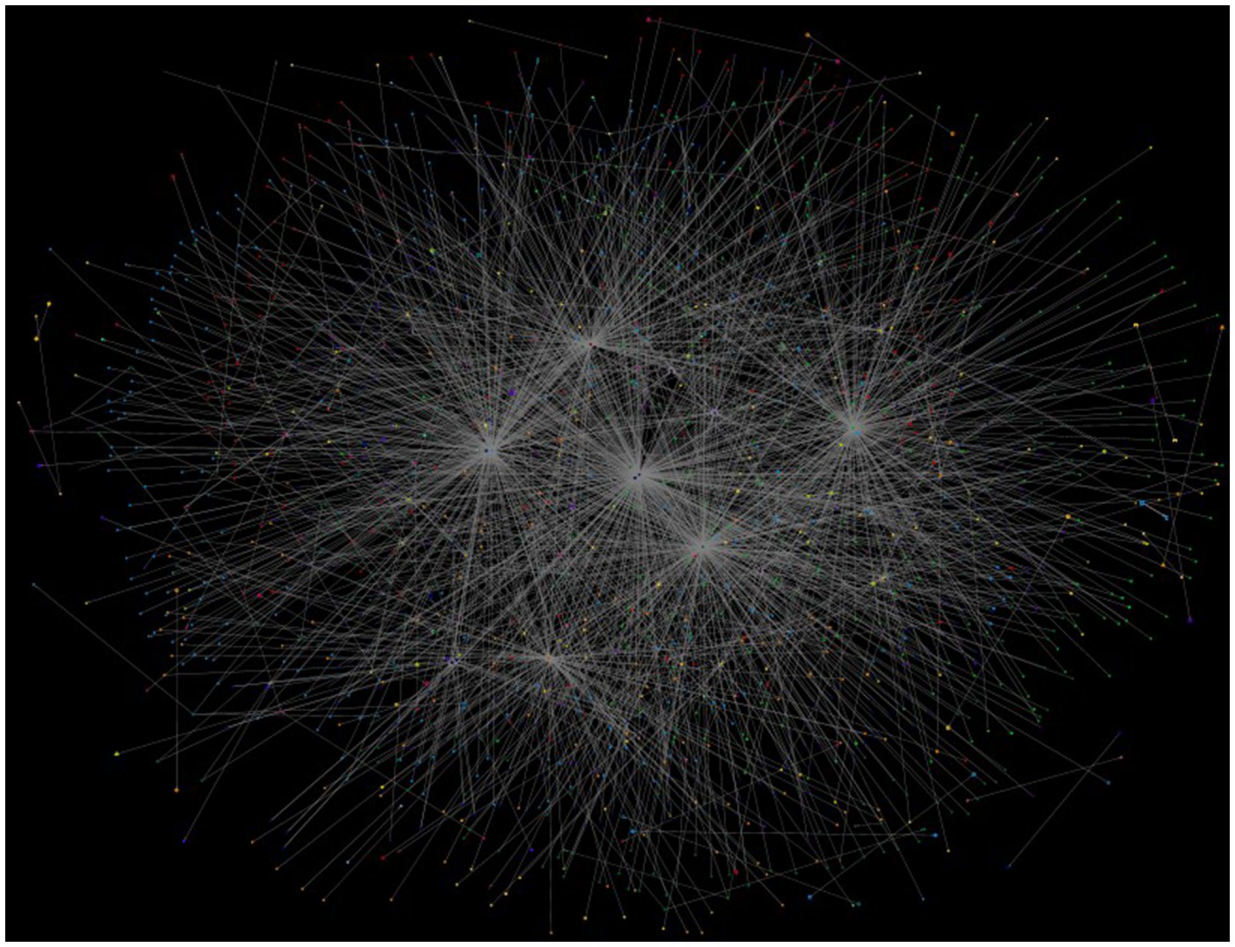
Entire social network map with #Syrian and #Earthquake. Each dot (vertex) represents a unique individual who contributed to this network with Twitter action including two hashtags, and the lines (edges) between the dots indicate Twitter actions(tweet, replies or mentions), *n*_user_ = 1918 and *n*_Twitter_action_ = 2,126.

There were 1918 Twitter users within our dataset. The dataset consisted of *n* = 274 tweets, *n* = 124 replies and *n* = 1726 mentions in total 2,126 Twitter action (Figure 1). The network had a high mentions percentage (81%), which indicates that Tweets on a certain topic are frequently discussed by users and influence the network more than others.

The variability in users’ contributions to the hashtag discussions was substantial, as indicated by the wide ranges in out and in degrees. High betweenness centrality, closeness, and eigenvector metrics shows that certain content and users had a greater influence on the overall network and were more effective in connecting different vertex (an individual who contributed to this network) than others (Table 1).

**Table 1:** Descriptive social network analysis metrics.

Metrics	Min	Max	Mean	Median
In-degree	0	323	1	0
Out-degree	0	27	1	1
Eigenvector	0	0,684	0,009	0,001
Closeness	0	0,254	0,114	0,154
Betweenness	0	819529,861	2826,077	0

**Figure 2:**
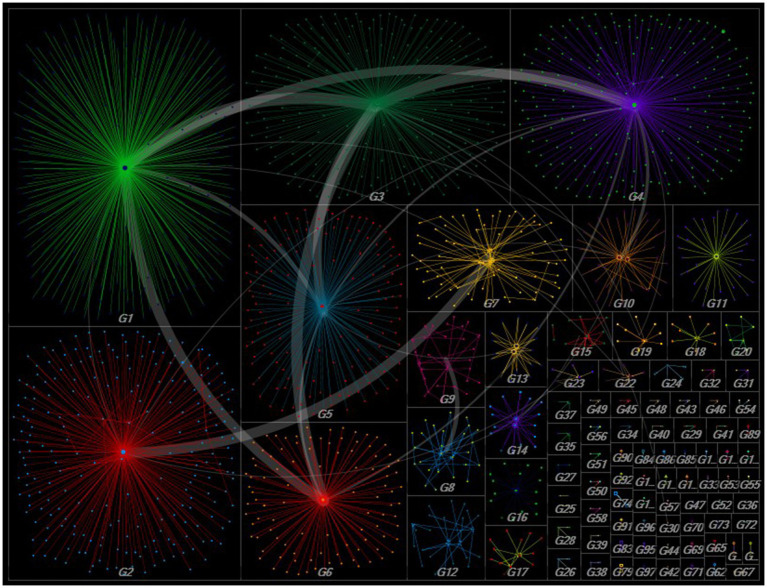
Social network graph clustered into group. Larger dots represent users who had a significant influence within the network, measured by the betweenness centrality metric and the bold lines delineate the dynamics of intergroup interactions within a given social network (*n*_groups(edges > 15)_ = 16).

The social network analysis conducted on the data uncovered that the conversations related to Syrian migrant and earthquake were formed across multiple groups. Figure 2 presents a graphical representation of how these sub-groups contributed to shaping the overall network and discussions revolved around the aforementioned topics. In total, there were 49 groups with more than three edges, out of which 16 groups were identified as having more than 15 edges. The larger dots in the figure represent users who had a significant influence within the network, measured by the betweenness centrality metric. The bold lines delineate the dynamics of intergroup interactions within a given social network. The network’s clustering analysis reveals the formation of groups centered on a particular tweet and its associated interactions, indicating the need for further investigation into which content is spread more than others in post-disaster environment.

Table 2 presents data on the top interacted tweets, which means that it has garnered some form of response or engagement from the Twitter community (retweeted, mentioned) and user characteristics of the first 10 groups sorted by vertex number which represents group members. Upon examining the originators of the tweets disseminated within the group, a diverse array of Twitter users can be observed, ranging from academics and journalists to government officials. In these groups, the tweets about Syrians in aftermath of the earthquake that were most frequently discussed centered around issues with border security and moving back to the country of origin. Some other Twitter users employ these two hashtags as a means of increasing their visibility using the country’s top topic agenda, even in the absence of any significant content. In particular situations, hashtags are used to deliberate upon the prevailing political agenda of the nation, particularly in relation to the ongoing electoral proceedings.

**Table 2:** Tweets and characteristics of users in the first 10 groups, ranked by vertex number.

Group	Vertex	Tweet	User	User chracteristics (self-statement)	Betweenness	In degree	Eigenvector
1	281	Turkey_[Türkiye]_ could have hindered the dreams of terrorist organization and the projects of countries that harbored the dream of the treaty of SEVR by sending Syrian immigrants back to their country. It should not be forgotten that some countries’ intelligence agencies especially follow migration movements after earthquakes…”	User 1	Academician, former military personnel	819529,861	323	0,684
2	236	It is being attempted to turn the issue of border security into a polemic with political considerations, personal ambitions, unrealistic, exaggerated, and misleading statements. Around 60,000 Syrian volunteers returned to their country after the earthquake. We will never allow any illegal crossings at the borders; such a thing is out of the question	User 2	Ministry of National Defense	675716,837	218	0,013
3	204	According to information from the Presidency of Migration Management, illegal Syrians and Afghans in the Repatriation Centers after the earthquake were released! They were required to leave the country within 30 days, under the pretext of “being relocated to earthquake victims.” The fate of those who did not leave is unknown	User 3	Journalist	514094,092	235	0,179
4	201	“What kind of game is being played through Syrians and illegal immigrants? According to information from the Presidency General of Migration Management, illegal Syrians and Afghans in the Repatriation Centers after the earthquake were released! They were required to leave the country within 30 days, under the pretext of “being relocated to earthquake victims.” The fate of those who did not leave is unknown! The practice of releasing with the condition of leaving the country within 30 days was already being done before the earthquake. The reason is the inadequacy of capacity in Repatriation Centers. Those who enter the country illegally are expected to leave the country of their own accord. There are thousands of undocumented immigrants among us! ‼ ‼ ”	User 4	Journalist- Economist	651104,172	195	0,085
4	201	Yesterday, I spoke with a Syrian and he said, “I will not go to Syria for the holiday, I’ll go after the elections,” and when I asked why, he said, “If current president leaves, they will send us back.” They say that they will register the earthquake-affected population in Hatay, and in order to use their votes, they say that all aid will be cut off if you register here	User 5	No information	602,291	11	0,011
5	138	The European Union is making efforts to orchestrate the settlement of Syrian refugees in the earthquake zone after the reconstruction of the area, instead of sending them back…	User 6	No information	357934,815	142	0,027
6	100	Same tweet in Group 3					
7	77	Same tweet in Group 2					
8	59	Three lawyers for each school are needed. The voter lists in houses, especially in areas where Syrian citizens and earthquake victims are located, in the earthquake zone, should be checked	User 7	Psychotherapist	6742,533	10	0,000
9	56	This *[a photo]* Syrian turned around with a knife and stabbed him in four places, including his heart, saying, “Wait, we will do very bad things to you,” after being warned for damaging a street light pole	User 8	Language expert	1050,000	7	0,000
10	44	A Syrian family who has been living in here for a year after the earthquake asked for help, saying, “I have six children. The youngest is 2, and the oldest is 12. There is no other way for us. We do not want anything. We just want a container for the children to live in…”	User 9	Engineer	51099,429	23	0,000

**Figure 3:**
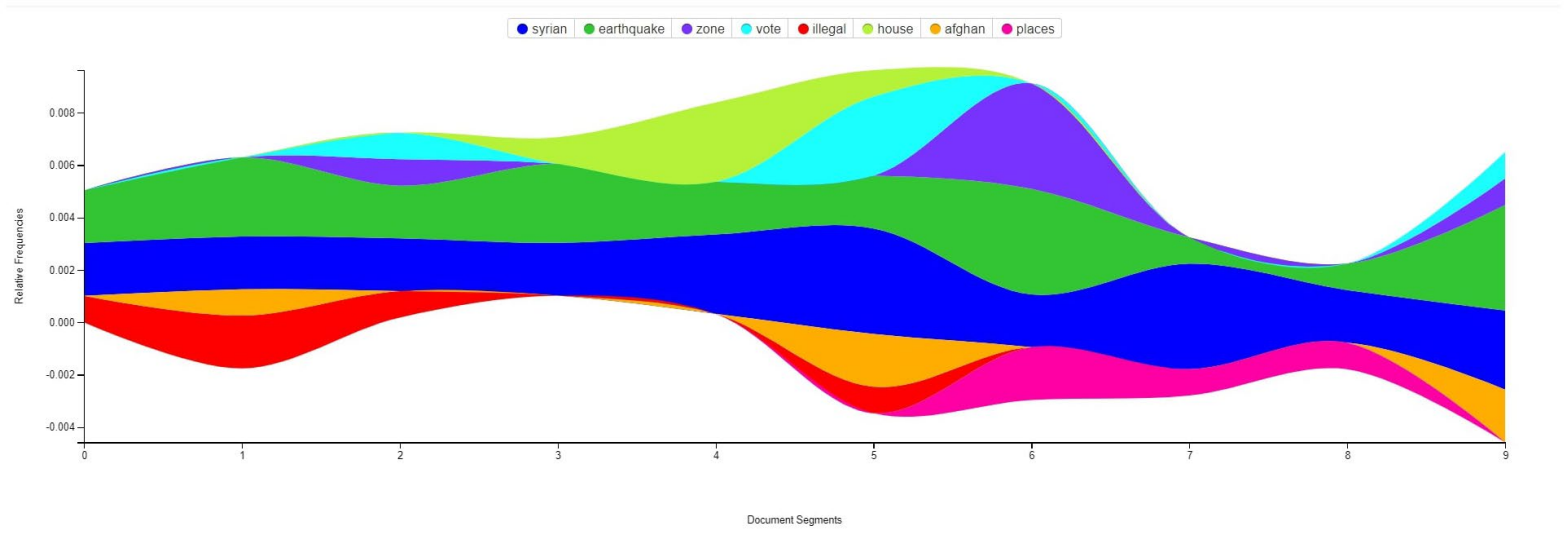
Stream Graph of the most frequently used words in the social network. Documented segments represents sequential parts of combined text of 27 tweets.

27 Tweets that were jointly decided as a data source to summative content analysis after extracting contents from the top-ranked groups, examining content in smaller groups and removing duplicates. The continuous presence of the analyzed terms shown in streamgraph (Figure 3), ‘Syrian’ and ‘earthquake’ throughout the combined tweet text data indicate the consistency of the content.

**Figure 4:**
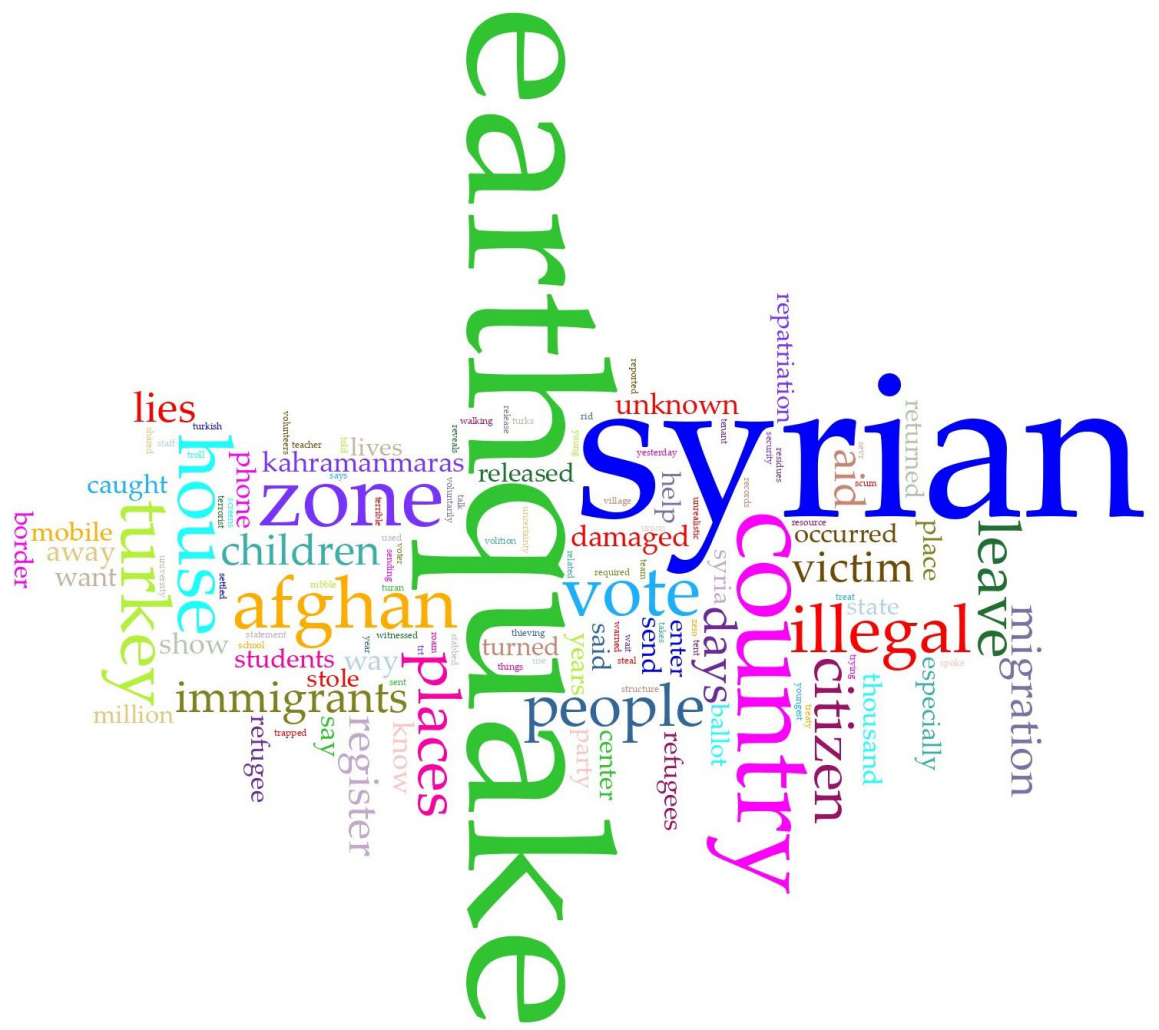
Word cloud analysis of tweets in the social network (*n*_tweet_: 27).

**Figure 5:**
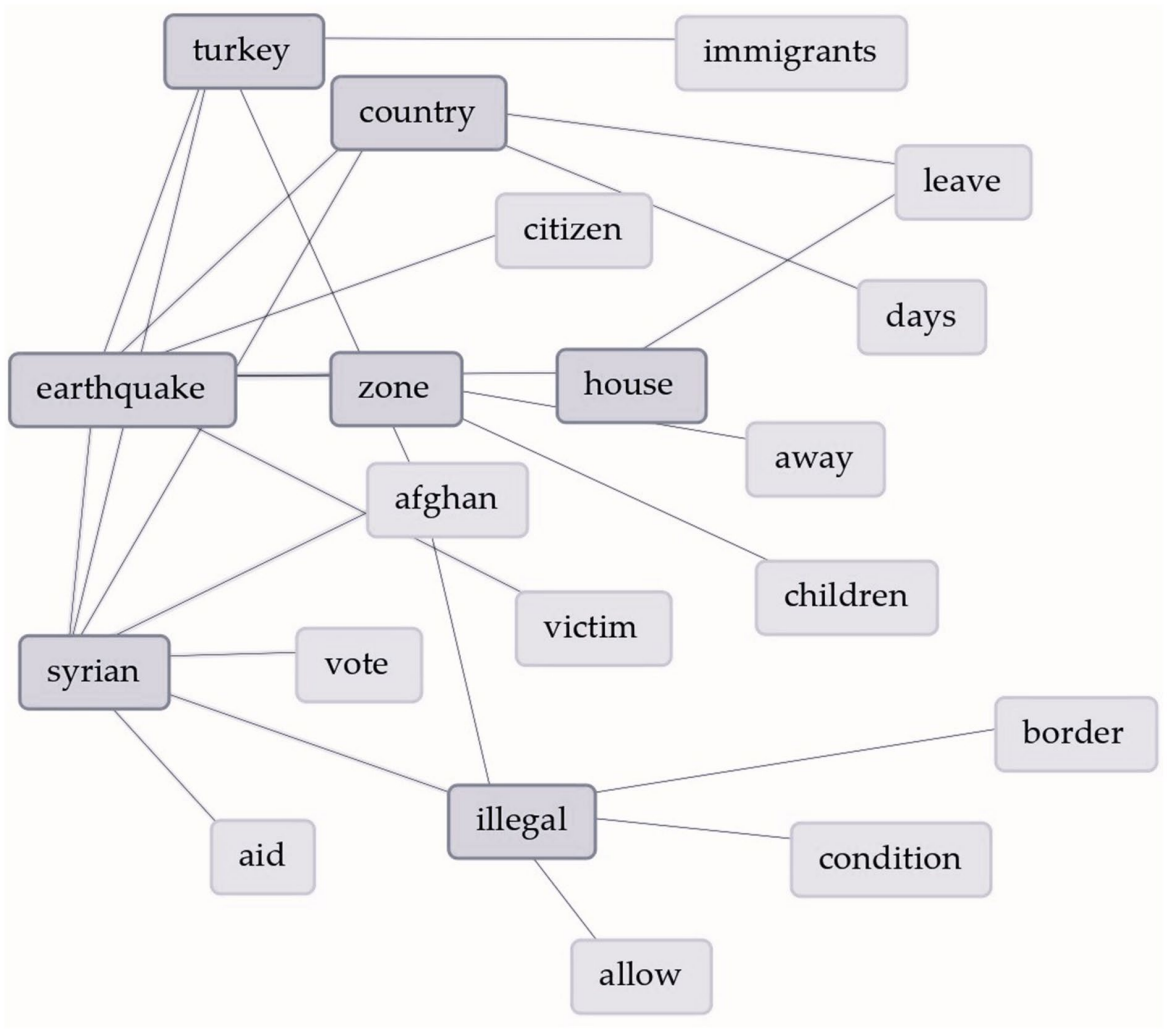
Word links graph of the most frequently used words and linkage.

Following word cloud analysis of all tweet data merged, Figure 4 depicts the most frequently used words. As anticipated, the designated hashtags ‘Syrian’ and ‘earthquake’ were most commonly observed within the dataset. The Word Links Graph (Figure 5) provides insights relationships of top 20 frequent words as a visual representation.

The most frequently used words are sorted into two, material-related and uncertainty-related by reading and re-reading of the entire content. After removing direct-material-related words as ‘Turkey_[Türkiye]_, earthquake, country, zone, Syrian, condition, days, away, immigrants, victim, allow’, most frequently used uncertainty-related words remained as *‘aid, vote, house, citizen, Afghan, illegal, children, border, and leave’*.

Tweets containing the aforementioned 9 words have been observed to address a range of main points, including but not limited to educational opportunities;

*“University opened up a lot of places for Syrian students, why don’t they take our children from the earthquake zone”*

double-sided concerns regarding the equitable allocation of aid resources;

*“If you pay attention, earthquake aid has benefited Syrians the most”*

*“They say if you register here, all aid will be cut off”*

and expressions of emotional reproach from a Syrian mother seeking housing for her children;

*“We don’t want anything. We just want a container for the children to live in…"*

or narratives about illegal migration;

“*How many Syrian and Afghan illegal immigrants entered Turkey_[Türkiye]_ after the earthquake?”*

Of the tweets pertained to uncertainty-related words, 9 main points were identified; being an undocumented migrant, concerns about deportation and housing, being or having a child, inequality of rights between being a citizen and non-citizen, being in a minority within a minority and access to education or generate new ones for migrants, such equitable distribution of aid, political climate of the host nation. With concentrating contemporary environment of the country and literature, the discussion section delved into a detailed explication of these main points which were identified as possible post-disaster uncertainties among migrants.

## Discussion

The research was designed to draw inferences from the content of collected tweets with hashtags ‘Syrian’ and ‘earthquake’ about the particular aspects of post-disaster environment which may have the potential to generate new or exacerbate pre-existing uncertainties encountered by migrants. Although social network analysis has certain limitations in its application to this topic, an analysis of community-generated content can still provide valuable insights into the matter under research. Representing the patterns of co-occurring words and discussions provides a deeper understanding of the phenomenon. It can also help identify trends and emerging topics, as well as provide insights into the language and discourse around a particular issue or event. Whilst knowing the limitations of generalizability, it is posited that the comprehension of intricate designs by multidisciplinary disciplines, such as public health will enhance the effectiveness of interventions within those.

The aftermath of a disaster poses unique challenges to those affected including migrants, who often experience heightened uncertainty and ambiguity in such environments. Apart from the uncertainties arising from the disaster *per se*, the post-disaster environment may give rise to fresh uncertainties by engendering new issues and reorganizing pre-existing ones, as political instability (50) and the legal status of undocumented migration (51). The uncertainties encountered by migrants exhibit a close connection with the political climate of the host nation, as shown by the tweets about significant political occurrences and with the most frequently used words as *‘vote and citizen’*. The combination of being both a Syrian migrant and a victim of the earthquake has rendered Syrian migrants a subject of political discourse, transforming their migratory identity into a political asset during Türkiye’s presidential and parliamentary election campaigns as *“Three lawyers for each school are needed. The voter lists in houses, especially in areas where Syrian citizens and earthquake victims are located, in the earthquake zone, should be checked.”* In this tweet, a user who suspects that Syrian migrants will vote unfairly in the earthquake zone, wants to assign lawyers to schools to prevent this. The statement pertains to the redefined status of migrants given the impact of prevailing political climate and disaster. The aforesaid dialogue is frequently observed in different shapes in social media and underscores the necessity for attention towards the influence of political occurrences on the uncertainty experienced by migrants in post-disaster environment.

The process of migration is inherently associated with a certain level of uncertainty (52). However, this uncertainty can be mitigated to a certain extent through the process of settling in the host country and gradually establishing a new daily routine (53). In instances where a catastrophic disaster occurs in the host nation, the rhetoric of deportation and related media coverage, including tweets and news mentioned the return of 42,000 migrants to their conflict-ridden homelands, might create uncertainty and confinement among migrants. Additionally, being an undocumented migrant can easily make individuals susceptible to the rising border security debates on social media, seen in our contents as “*How many Syrian and Afghan illegal immigrants entered Turkey_[Türkiye]_ after the earthquake?”* and lead them to further uncertainty. This circumstance may easily impose a mental and social burden on health, affecting all aspects of well-being. The words *‘illegal, border, and leave’* frequently used in social media analysis invite us to conduct detailed research and stay vigilant in this regard.

The difficulties that migrants experience in their new country, including having challenges in getting access to housing (54) and education (55, 56), are already demanding and cast uncertainty on their future. These uncertainties are further amplified by the internal population movements caused by the earthquake, which disrupted the current dynamics of cities and regions, and pauperized the affected citizens and migrants to the same needs. A discernible attitude of competing interests arises between citizens and migrants, as seen by social media contents linked with the most frequently used word *‘house’*, involving citizens expressing unhappiness with the presence of migrants as tenant *“She_[a Turkish citizen]_ lost her house due to the earthquake and now she lives in a tent. She bought another house in the neighborhood. There is a Syrian tenant residing inside. And the Syrian tenant refuses to show her the home or leave the house. Is this how we treat our citizen in this country?” and* Syrian migrants seeking housing *“We do not want anything. We just want a container for the children to live in….”* The presented contents provide findings for the argument that housing has the potential to be a significant contributor to post-disaster uncertainty. It is important to stress that the absence of essential rights, such as housing, which serve as crucial social determinants of health (57), alongside the psychological burden stemming from the uncertainty (58) of being deprived of these rights, must be considered in interventions to promote migrant’s health.

The recurrent use of the word ‘children’ prompted us to examine diverse sub-fractions of vulnerability in migration status. In addition, the post-disaster environment highlights that vulnerability is not an unilayered structure (59, 60). The concept of ‘vulnerable of vulnerable’ holds significant relevance in the context of migrant children in this research, given its comprehensive coverage of the complex ramifications associated with being a migrant, dealing with disaster, and being a child. As evidenced by numerous studies, being a migrant child entails a plethora of negative consequences (61, 62). The visibility of these negative impacts of being a migrant child is reflected in tweets ranging from a migrant mother seeking housing for her children to criticism of the some of the limited school quotas opened for Syrian migrant children and youth; *“University opened up a lot of places for Syrian students, why do not they take our children from the earthquake zone”*. This inference serves as a useful reference point to capture the layered nature of vulnerability and indicates the need for nuanced and intersectional approaches in addressing the needs of vulnerable populations.

This research is also significant in terms of supporting the ongoing –unpublished doctoral thesis of the researcher, in which sub-uncertainty themes including the loss of life and even unknown country of the deceased’s burial are identified in the qualitative research part. In the post-disaster context, it has been observed that nearly half of Syrian migrants who lost their lives were buried in Türkiye, while the other half were sent to Syria for burial (8, 9). For instance, this research aligns with the preliminary analysis in the quantitative section of this thesis, which found that migrant women experience more uncertainty than men. This is consistent with the media-visibility of a mother’s concerns for her children.

Another confrontation arises between a minority (Afghans) within a minority (Migrants) and a majority (Syrians) within a minority (Migrants). A few tweets exhibit the utilization of hashtags, such as ‘Syrian’ and ‘Afghan’ in conjunction, emphasizing an idea that even within a minority group, particular hashtags have a hierarchical level of popularity in their usage. The aforementioned punctuates the intricate nature of social identities and the stratified presence of visibility within the realm of digital media and underscores different power dynamics appeared in the physical world and resulting climate of uncertainty.

The frequent use of the word *‘aid’* provides valuable insights into both uncertainty and humanitarian aid, with the equitable distribution of aid being identified as a source of uncertainty in post-disaster environment, as seen in such Tweets, *“They say if you register here, all aid will be cut off” and “If you pay attention, earthquake aid has benefited Syrians the most.”* It may be difficult to ensure that aid reaches those who need it most due to high demand for resources and services, which can cause social tensions to rise and feelings of unfairness. Transparent and equitable aid distribution mechanisms are necessary to foster trust and social cohesion (63, 64). This would guarantee that funds are distributed based on need, not prejudice or preference, enabling successful recovery and reconstruction operations. The equitable distribution of humanitarian aid holds paramount importance in post-disaster environment due to the potential of unequal access to further marginalize vulnerable communities.

After disasters, the sense of destruction and uncertainty can induce panic and uncontrollable behavior (65). Besides, studies have indicated that looting have a tendency to escalate in the aftermath of a disaster (66). This can contribute to a decrease in tolerance levels and an increase in hate speech as seen in social media “*How many refugees (Afghan, Syrian*, etc.*) caught while looting should be deported together with their families*.” Unverified accusations directed towards migrants through social media and other circulated media content have power to influence the perceptions of the host community to migrants and exacerbate uncertainty experienced by migrants. This phenomenon could lead to increased levels of aggression within the host community. A post-disaster environment has the potential to result in increased social tensions, reduced tolerance levels, and triggered violence (67), a fundamental public health problem (68) that warrants focus.

In this research, analysis of social media was utilized to indirectly explore the potential contribution of post-disaster environment to uncertainty. The purpose of the research is not to scrutinize the uncertainty engendered by social media or the ways in which uncertainty is articulated through social media, but rather to highlight the beneficial role of research based on social media utilization in comprehending uncertainty. However, it is essential to recognize that social media itself can play a role in generating uncertainty in various ways, particularly in crisis settings (69). Social media platforms offer individuals a virtual arena to share thoughts, emotions, and experiences, particularly during disasters, such as earthquakes. Social media become a hub for seeking and sharing information, expressing concerns, and connecting with others. Users might express uncertainty through ambiguous language, open-ended questions, and seeking advice or reassurance (44, 69, 70). Understanding how uncertainty is expressed, experienced and generated on the digital platforms provide insight into the wider societal and psychological intersections of the post-disaster environment as a further research topic.

Contrary to our main finding which shows the destructive language of social media regarding migrants in post-disaster environment, social media also take on many supportive tasks in times of disaster. It plays a significant role in promoting self-resilience during times of disasters by facilitating information exchange and creating extensive networks for gathering useful information (71). With its high information capacity, reliability, and interactive potential, social media offer advanced disaster communication opportunities (72). Moreover, they serve as a valuable resource for crisis management following disasters (73). During emergency situations, social media are recognized as a crucial tool for responders and decision-makers, as they provide situational and actionable information through text messages and shared images (74). These characteristics of social media contribute to enhancing disaster response efforts and enabling effective communication among affected individuals, emergency responders, and the wider community. The timely and accessible nature of social media content allows for rapid dissemination of critical information, aiding in the coordination of relief efforts and facilitating community resilience. Therefore, further research should be conducted to investigate whether the language used on social media in the post-disaster context represents a discourse directed towards migrants, if it is merely a reflection of the data collection period in this research, or if it holds significant representational potential.

These arguments focus attention on the significance of verifying news and content, developing media literacy, and evaluating social media content in the context of infodemic management. The velocity and influence of data associated with prevalent hashtags, as well as the perturbed dissemination of information, necessitate meticulous evaluation. The careful consideration of the impact of generated content is particularly crucial in sensitive environments, such as post-disaster environment. The results of our cluster analysis indicate that certain individuals possess a considerable influence over extensive audiences, and these individuals ought to refrain from disseminating content that could negatively impact the well-being of migrants. The appropriate use of social media can help in addressing the uncertainties posed by post-disaster environment, rather than exacerbating. This approach displays substantial promise in propelling two interconnected fields that have been coevolving in recent pandemic era (75): public health and infodemic management.

### Limitations

There are a number of limitations in this research, including the utilization of Turkish tweets with two main hashtags for analysis and the implementation of an indirect perspective as part of the research design. The lack of inclusion of relevant Turkish hashtags associated with disasters such as emergency, need, and help, may have resulted in the omission of significant tweets, thereby posing a potential risk of being overlooked. It is imperative to meticulously contemplate this aspect in forthcoming research endeavors to guarantee thorough scrutiny of the subject matter. The analysis was conducted using a time-constrained sample of Twitter data, which may limit the generalizability of the findings to broader population. The data were gathered at the designated time with the intent of circumventing the information overload that transpired during the primary acute stage of the earthquake. It is likely that alternative patterns pertaining to the phenomenon would have surfaced, if the data had been gathered during a distinct period. Additionally, since Twitter APIs have been restricted to open access, data collection had to be kept shorter than originally planned. Furthermore, because social media are not always available to all people, the research might have overlooked crucial viewpoints from people who do not have access to social media. Notwithstanding the aforementioned limitations, this research provides valuable perspectives on the probable impetuses of post-disaster uncertainty among migrants and underscores the necessity of interdisciplinary methodologies to tackle the challenges encountered by these groups.

## Conclusion

This research conducted an analysis of social media data in order to gain insight into the potential of the post-disaster environment generating new uncertainties or exacerbating pre-existing ones for migrants in the context of ‘Türkiye-2023 Earthquake’ and ‘Syrian migrants’. Through content and network analysis, we highlighted the importance of discussing current events, relevant sociopolitical issues, and literature to identify the main points of the tweets as possible post-disaster uncertainties among migrants. Our findings emphasize that the post-disaster environment has potential to exacerbate existing uncertainties, such as being an undocumented migrant, concerns about deportation and housing, being or having a child, inequality of rights between being a citizen and non-citizen, being in a minority within a minority, political climate of the host nation and access to education or generate new ones for migrants such equitable distribution of aid which can lead to poor health outcomes. Ultimately, recognizing the possible uncertainties in post-disaster environment among migrants and addressing the probable underlying factors might help to build more resilient and healthy communities.

## Data availability statement

The raw data supporting the conclusions of this article will be made available by the authors, without undue reservation.

## Author contributions

GA: conducting research question and methodology, SNA data analysis, qualitative conceptualization, writing original paper. ŞB-Ö: qualitative conceptualization, second review and editing. All authors contributed to the article and approved the submitted version.

## Conflict of interest

The authors declare that the research was conducted in the absence of any commercial or financial relationships that could be construed as a potential conflict of interest.

## Publisher’s note

All claims expressed in this article are solely those of the authors and do not necessarily represent those of their affiliated organizations, or those of the publisher, the editors and the reviewers. Any product that may be evaluated in this article, or claim that may be made by its manufacturer, is not guaranteed or endorsed by the publisher.
